# 1-Nitro-2-phenylethane: a promising phytoconstituent to modulate neuroinflammation and oxidative stress with repercussions on neurological and psychiatric disorders

**DOI:** 10.3389/fphar.2025.1552295

**Published:** 2025-05-02

**Authors:** Lucas Villar Pedrosa Silva Pantoja, Emily Christie Maia Fonseca, Fábio José Coelho Souza-Junior, Brenda Costa da Conceição, Sarah Viana Farias, Joyce Kelly R. da Silva, José Guilherme Soares Maia, Pedro Iuri Castro Silva, Jofre Jacob Silva Freitas, Rui Daniel Prediger, Daniele Luz Campos, Enéas Andrade Fontes-Júnior, Cristiane Socorro Ferraz Maia

**Affiliations:** ^1^ Laboratório de Farmacologia da Inflamação e do Comportamento, Instituto de Ciências da Saúde, Universidade Federal do Pará, Belém, Para, Brazil; ^2^ Laboratório de Biotecnologia de Enzimas e Biotransformações, Universidade Federal do Pará, Belém, Para, Brazil; ^3^ Programa de Pós-Graduação em Ciências Farmacêuticas, Universidade Federal do Pará, Belém, Para, Brazil; ^4^ Centro de Estudos Pré-Clínicos da Amazônia, Universidade do Estado do Pará, Belém, Para, Brazil; ^5^ Laboratório Experimental de Doenças Neurodegenerativas, Departamento de Farmacologia, Centro de Ciências Biológicas, Universidade Federal de Santa Catarina, Florianópolis, Santa Catarina, Brazil

**Keywords:** 1-Nitro-2-phenylethane, Aniba canelilla, Neuroinflammation, oxidative stress, psychiatric illness

## Abstract

Neurologic and neuropsychiatric disorders are complex, with common pathophysiological mechanisms associated with inflammation, oxidative stress, and vascular damage. These shared features have stimulated interest in bioactive compounds with neuropharmacological potential. In this regard, the 1-Nitro-2-Phenylethane (1N2PE) emerges as a promissory compound to act on the multiple via of brain disturbances. However, its neuropharmacological mechanisms of action remain largely unknown. We aim to provide a comprehensive overview of the scarce literature on the effects of 1N2PE in brain disorders to highlight the importance of further research into the mechanisms of action and its potential applications in the field of neurology and psychiatry, focusing on the anti-inflammatory and antioxidant properties. The 1N2PE exhibits neuroprotective properties, including anti-inflammatory, antioxidant, and cholinergic-enhancing effects, which together may underlie its potential therapeutic benefits for various neuropsychiatric and neurological disorders, such as depression, anxiety, seizures, and cognitive impairments. This review compiles literature on 1N2PE potential central nervous system activities, highlighting its therapeutic potential in treating behavioral and neurological disorders. Despite promising findings, further research is essential to fully understand 1N2PE as a novel therapeutic agent.

## 1 Introduction

Folk medicine is a primary therapeutic approach in underdeveloped countries, which the use of a variety of natural products, including medicinal plants (i.e., phytotherapy) plays a pivotal role (Rizvi et al., 2022; da Silveira et al., 2016; Menezes da Silveira et al., 2021). Medicinal plants also serve as reservoirs of bioactive compounds that exert significant pharmacological actions. For example, syringic acid (SA), a benzoic acid derivative obtained from *Isatis indigotica* (Cikman et al., 2015), *Capparis spinosa* L. (Turgut et al., 2015), and other sources, has demonstrated various neuroprotective effects by modulating neuroinflammation and oxidative balance, protecting neuronal cells by reducing the production of pro-inflammatory cytokines and reactive oxygen species (ROS), becoming a promising compound to act on neurodegenerative diseases (Ogut et al., 2022; Güzelad et al., 2021).

In this context, 1-nitro-2-phenylethane (1N2PE) emerges as another bioactive benzoic compound, distinguished by its rare nitro group, yet remains underexplored despite its valuable ethnopharmacological attributes (de Campos et al., 2023). Plants containing 1N2PE have a profound impact on traditional medicine in regions such as Africa and South America (Daï et al., 2022). For instance, Dennetia tripetala and Uvaria chamae are widely used in African countries to treat central nervous system (CNS) disorders (Daniyan et al., 2021; Daï et al., 2022). Similarly, in the Brazilian Amazon, *Aniba canelilla* is a cornerstone of traditional medicine, underscoring the therapeutic significance of 1N2PE in regional practices (Souza-Junior et al., 2020).

Considering central nervous system (CNS) diseases, the World Health Organization (WHO) reveals that about one in eight people globally live with a mental disorder. Consequently, psychiatric disorders have emerged as a global public health concern of high interest, requiring increased attention due to their negative impact on social functioning and overall quality of life (World Health Organization, 2022b). Some neurologic conditions, such as dementia, also represent a critical CNS disorder characterized by severe neurodegeneration, leading to cognitive and behavioral deficits, which in turn result in psychiatric illness (World Health Organization, 2022a). In this context, it is well documented the role of neuroinflammation in the pathophysiology of CNS disorders (Thomson and Craighead, 2008; Troubat et al., 2020; Wu and Zhang, 2023; Guo et al., 2023). Nevertheless, the primary therapeutic approach for such conditions typically involves the modulation of neurotransmitters, which a significant portion of patients remain refractory to current pharmacotherapy (Strawbridge et al., 2015; Haroon et al., 2018; Osimo et al., 2020; Cui et al., 2024). This reality highlights the urgent need to explore novel therapeutic agents, such as bioactive molecules from medicinal plants. For instance, nitrocompounds exhibit various pharmacological approaches, such as antibacterial, antifungal, antihypertensive, antidepressant, and anxiolytic activities (Olender et al., 2018; Nepali et al., 2018; Sukhorukov, 2020; Noriega et al., 2022; Abreu et al., 2024). Therefore, a significant group of botanicals present nitrocompounds as constituents in their compositions (Noack, 1991; Divakaran and Loscalzo, 2017; Sadeghpour et al., 2017; Müller et al., 2019; Carradori et al., 2018; Olender et al., 2018).

Previously, our research group demonstrated that *Aniba canelilla*, a plant rich in 1N2PE, exhibits robust biological activities, particularly related to antinociceptive effects, cardiovascular modulation, attenuation of inflammation and oxidative stress, and cognitive-enhancing effects (Souza-Junior et al., 2020; de Campos et al., 2023; Cardoso et al., 2022). Despite these findings, there is a significant gap in the literature regarding the neuropharmacological properties of *Aniba canelilla* and its key biomarker, 1N2PE. Anti-inflammatory and antioxidant activities have been attributed to 1N2PE (Cardoso et al., 2022). Considering the pivotal role of the shared neuroinflammation-oxidative stress feedback loop in the pathogenesis of numerous neuropsychiatric and neurological disorders, we hypothesize that the modulation of inflammatory and oxidative pathways constitutes a critical mechanism underlying the neuropharmacological effects of 1N2PE. Thus, the present review has three main objectives: (1) to compile and analyze the existing ethnopharmacological literature on the effects of 1N2PE, particularly its role on neurological and psychiatric disorders; (2) to identify and discuss putative molecular targets of 1N2PE that support its pharmacological activities, with a focus on its modulation of neuroinflammation and oxidative stress mechanisms; and (3) to provide an integrative perspective on the 1N2PE bioactivity that aligns with the pathophysiological processes underlying neuropsychiatric and neurological conditions. This comprehensive approach aims to bridge the knowledge gap in the neuropharmacological knowledge of 1N2PE and to highlight its potential as a therapeutic agent in the treatment of psychiatric and neurological conditions.

## 2 Methodological aspects

A comprehensive search was conducted across multiple scientific databases, including PubMed, Cochrane, Web of Science, and Scopus, to identify studies related to 1N2PE. The search employed the following terms: (“1-Nitro-2-phenylethane” OR 1N2PE OR “(2-Nitroethyl) benzene” OR “2-Nitroethylbenzene” OR “1-(2-nitroethyl) benzene” OR “2-phenylnitroethane” OR “2-nitro-ethyl-benzene” OR “1-phenyl-2-nitroethane”). The selection criteria included original and review articles that explored the neuropharmacological and ethnopharmacological properties of 1N2PE. No language restrictions were applied to ensure a comprehensive inclusion of relevant studies. The search process was performed independently by two researchers, who evaluated the articles based on predefined inclusion criteria. In cases of disagreement regarding article inclusion, a third experienced researcher was consulted to reach a consensus.

## 3 Ethnopharmacological properties of 1N2PE-rich plants related to CNS disorders

Traditional medicine emerges as a powerful tool to treat illnesses, especially within local communities in developing countries (Rizvi et al., 2022). It is important to highlight the rarity of 1N2PE, since few plants contain such compound. The primary sources of 1N2PE include *Aniba canelilla* (native of Brazil), *Ocotea pretiosa*, *Dennetia tripetala*, and *Uvaria chamae* (native of African countries). Interestingly, these medicinal plants originate from regions with strong traditions of traditional medicine, which cultural aspects combine to offer valuable alternatives for disease treatment.

To date, there are few scientific reports focusing on the ethnopharmacological properties of *Ocotea pretiosa*. However, it has been reported the stimulant, diuretic, carminative, analgesic, rubefacient, and blood-purifying activities (for review see Souza-Junior et al. (2020)).

*Dennetia tripetala* has been well-documented for its use in treating CNS disorders, such as anxiety, amnesia, epileptic seizures, and pain (Oyemitan et al., 2013; Souza-Junior et al., 2020). Furthermore, this plant exhibits numerous other pharmacological effects, including antioxidant and anti-inflammatory effects, which contribute to the CNS benefits. Similarly, *Uvaria chamae*, although less known, is equally relevant in African ethnomedicine, particularly for its neuropharmacological activities (Aiyeloja and Bello, 2004; Catarino et al., 2016). (Dai et al., 2022) conducted a robust bibliometric review highlighting the significance of this plant in traditional Nigerian medicine, especially for the treatment of mental disorders and neurological diseases.

*Aniba canelilla*, known as *casca-preciosa* (precious bark), *falsa-canela* (false cinnamon), and *folha-preciosa* (precious leaf) among Amazonian communities, has been used traditionally for a variety of sickness, including gastrointestinal disorders, ulcers, colds, coughs, nausea, dermatitis, pain, anemia, and infections (Souza-Junior et al., 2020). Additionally, its essential oil has been used for “calming effects” and for the potential to alleviate symptoms associated with neuropsychiatric conditions. Ethnopharmacological studies suggest that 1N2PE exhibits significant anti-inflammatory and antioxidant properties, which may contribute to its neuroprotective effects, making this compound a valuable candidate for further research into CNS disorders. Thus, its traditional uses encompass analgesic, anti-inflammatory, antioxidant effects, and in the treatment of CNS disorders, such as Alzheimer’s disease and depression (Maia et al., 2001; De Filipps et al., 2004; Taylor, 2005; de Lima et al., 2009; Barbosa et al., 2017; Souza-Junior et al., 2020).

Based on the traditional use of botanicals that present the 1N2PE, pharmacological studies were conducted to investigate the biological potential of 1N2PE and confirm its anti-inflammatory, antinociceptive, antihypertensive, antioxidant, anticonvulsant, anxiolytic, and cognitive effects (Souza-Junior et al., 2020; de Campos et al., 2023). Such initial findings suggest the potential of this nitrocompound to treat CNS disorders. However, despite these findings, studies on the central activities of 1N2PE are still scarce.

## 4 Distribution and natural sources of 1-Nitro-2-phenylethane: role of Amazon biome

1N2PE was first identified and isolated in two Lauraceae species: *Ocotea pretiosa* (Nees and Mart.) Mez from South/Southeast regions and *Aniba canelilla* (Kunth) Mez from the brazilian Amazon (Gottlieb and Magalhães, 1959). Additionally, 1N2PE has been identified in *Dennettia tripetala* Baker f. (Annonaceae), *Uvaria chamae* P. Beauv. (Annonaceae), and *Stephanotis floribunda* Brongn. (Apocynaceae) from the African continent, as well as *Eriobotrya japonica* (Thunb.) Lindl. (Rosaceae) from China (Adeoti et al., 2000; Pott et al., 2002; Owolabi et al., 2013; Kuwahara et al., 2014). 1N2PE was also identified in tomato flavors by sensory and instrumental analysis of existing cultivars at the University of Florida, USA (Baldwin et al., 1998).

It is noteworthy that the Amazon species *Aniba canelilla* is the primary source of 1N2PE. The pleasant cinnamon-like odor is attributed to the meaningful content of 1N2PE in the plant’s trunk and bark wood (Gottlieb and Magalhães, 1959). It is well established that the composition percentage of phytochemical compounds in a plant matrix depends on some variables associated with seasonality (Ramasar et al., 2022). Previous literature postulated that differences in the 1N2PE content from leaves, wood, bark, and flowers of *Aniba canelilla* occur in distinct collections sites and year seasons, confirming that the seasonality influences the percentual of secondary metabolites (Gottlieb and Magalhães, 1959; 1960; Oger et al., 1994; Villegas et al., 1997; Taveira et al., 2003; Lima et al., 2004; Lahlou et al., 2005; da Silva et al., 2007; Silva et al., 2009; de Lima et al., 2009; Sousa et al., 2009; de Siqueira et al., 2010; Manhães et al., 2012; Silva et al., 2014; Barbosa et al., 2017; Giongo et al., 2017; Kreutz et al., 2021; Cardoso et al., 2022; de Campos et al., 2023). The literature reveals that the 1N2PE exhibits a high percentage in the composition of *Aniba canelilla* oil, which characterizes this aromatic species (Table 1).

**TABLE 1:** Primary sources of 1-nitro-2-phenylethane (1N2PE) in essential oils of aromatic plants.

Country	Collection sites	Species	Plant parts	Oil %	1N2PE %	References
Brazil	Atlantic and Amazon Forests	*Ocotea pretiosa* *Aniba canelilla*	Wood/Bark Wood/Bark	0.1/0.1 0.7/0.6	n.m 80	Gootlieb and Magalhães (1959), Gootlieb and Magalhães (1960)
Bolivia	Beni Department	*Aniba canelilla*	Bark	n.m	89.8	Oger et al. (1994)
Brazil	São Paulo	*Aniba canelilla*	Bark	0.1	71.1	Villegas et al. (1997)
Africa	Ketore, Benin	*Dennettia tripetala*	Leaves	0.2	53.7	Adeoti et al. (2000)
Africa	Madagascar	*Stephanotis floribunda*	Flowers	SPME	31.3	Pott et al. (2002)
Brazil	Carajás, PA, Mn mine Carajás, PA, Cu mine Carajás, PA, Urban area	*Aniba canelilla*	Leaves Bark Wood	0.5^a^ ^,^ ^b^ 0.9^a^; 0.8^b^ 0.6^a^; 05^b^	70.6^a^; 39.0^b^ 94.3^a^; 48.6^b^ 70.0^a^; 47.5^b^	Taveira et al. (2003)
Brazil	Carajás, PA, Cu mine	*Aniba canelilla*	Leaves Bark Wood	0.8^a^; 0.8^b^ 0.7^a^; 0.8^b^ 0.5^a^; 0.6^b^	94.5^a^; 39.3^b^ 87.1^a^; 56.2^b^ 80.4^a^; 53.3^b^	Taveira et al. (2003)
Brazil	Carajás, PA Urban area	*Aniba canelilla*	Leaves Bark Wood	0.7^a^; 0.8^b^ 0.8^a^; 0.9^b^ 0.7^a^ ^,^ ^b^	95.3^a^; 42.1^b^ 78.2^a^; 68.1^b^ 69.2^a^; 73.3^b^	Taveira et al. (2003)
Brazil	Paragominas, PA, Cauaxi River	*Aniba canelilla*	Bark	1.0	52.4	Taveira et al. (2003)
Brazil	Manaus	*Aniba canelilla*	Leaves Fine stems	0.8 0.2	71.2 68.2	Lima et al. (2004)
Brazil	Cauaxi River, Paragominas, PA	*Aniba canelilla*	Bark	1.0	95.0^a^; 39.0^b^	Lahlou et al. (2005)
Brazil	Novo Airão, AM	*Aniba canelilla*	Leaves Wood	1.3 0.2	91.8 92.1	da Silva et al. (2007)
Brazil	Ulianópolis, PA	*Aniba canelilla*	Leaves Wood Bark	1.2 0.2 0.5	74.0 70.2 90.3	da Silva et al. (2007)
Brazil	Manaus, AM	*Aniba canelilla*	Leaves	1.3^a^; 1.2^b^	88.5^a^; 88.9^b^	Silva et al. (2009)
Brazil	Cauaxi River, Ulianópolis and Paragominas, PA	*Aniba canelilla*	Bark Bark	0.6 1.1	71.0 52.9	de Lima et al. (2009) Sousa et al. (2009), de Siqueira et al. (2010)
Brazil	Cauaxi River, Paragominas, PA	*Aniba canelilla*	Bark	1.1	52.9	Sousa et al. (2009)
Brazil	Itacoatiara, AM	*Aniba canelilla*	Leaves Fine stems	0.3 0.3	52.2 92.7	Manhães et al. (2012)
Africa	Nigeria	*Dennetia tripetala*	Dried seeds	5.8	93.6	Oyemitan et al. (2013)
Africa	Nigeria	*Uvaria chamae*	Leaves	0.8	63.2	Owolabi et al. (2013)
Brazil	Cauaxi River, Ulianópolis, PA	*Aniba canelilla*	Wood	0.5	70.2	Silva et al. (2014)
Japan	Kyoto	*Eriobotrya japonica*	Flowers	SP Trap	32.6	Kuwahara et al. (2014)
Brazil	Itacoatiara, AM	*Aniba canelilla*	Leaves Branches	n.m n.m	31.2–84.3^a^ 13.2–74.6^b^ 90.8–93.6^a^ 87.9–94.2^b^	Barbosa et al. (2017)
Brazil	Itacoatiara, AM	*Aniba canelilla*	Wood	0.6	83.7	Giongo et al. (2017)
Brazil	Itacoatiara, AM	*Aniba canelilla*	Bark	0.5	86.6	Kreutz et al. (2021)
Brazil	Belém, PA	*Aniba canelilla*	Leaves	1.3	77.5	Cardoso et al. (2022)
Brazil	Ulianópolis, PA	*Aniba canelilla*	Wood	0.5	76.2	de Campos et al. (2023)

^a^ Rainy season (March/April).

^b^ Dry season (October/November).

Obs.n.m = not mentioned.

Remarkably, the uniqueness of 1N2PE and the fact that this uncommon constituent has been identified in the essential oil of *Aniba canelilla*, a species native to the Amazon Biome, highlights the importance of this region as an inexhaustible source of natural products for therapeutic purposes.

## 5 Chemical properties

**FIGURE 1:**
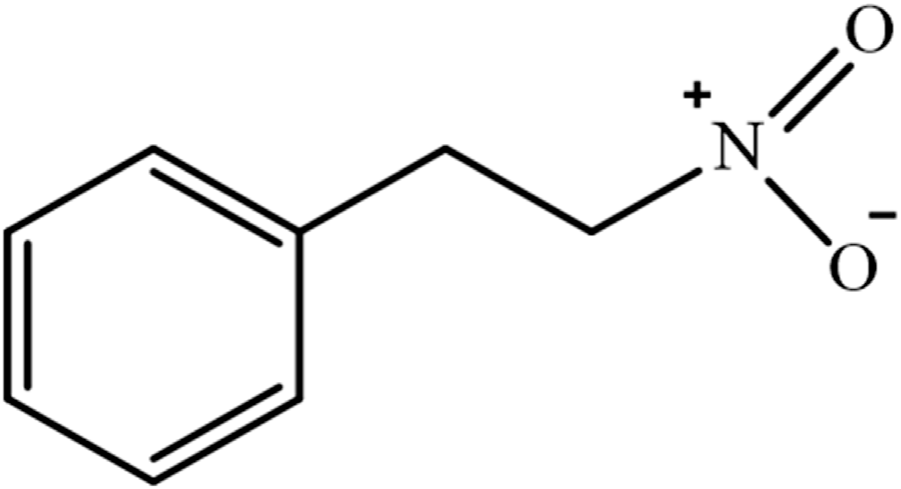
Chemical structure of 1-nitro-2-phenylethane (1N2PE).

Biosynthetically, the 1N2PE is a biologically active compound obtained by the biotransformation of phenylalanine by cytochrome P450 enzymes (CYP 450), considered the first nitro-derived compound found in plants. Due to its characteristic odor of cinnamon, similar to other constituents of the secondary metabolism of species popularly known as “canela”, the botanic species that exhibits the 1N2PE have been designated as “canela-sassafras” [cinnamon-sassafras, *Ocotea odorifera* (Vell.) Rohwer, syn. *Ocotea pretiosa* (Nees) Mez] and “falsa-canela” [false-cinnamon, *Cinnamomum cassia* (L.) J. Presl] (Gottlieb and Magalhães, 1959; Gottlieb and Magalhães, 1960; Matsuo et al., 1972; Almeida et al., 2017; Souza-Junior et al., 2020) (Figure 1).

## 6 Inflammation and oxidative stress in the spotlight: role of 1-Nitro-2-phenylethane on neuropsychiatric and neurological disorders

**FIGURE 2:**
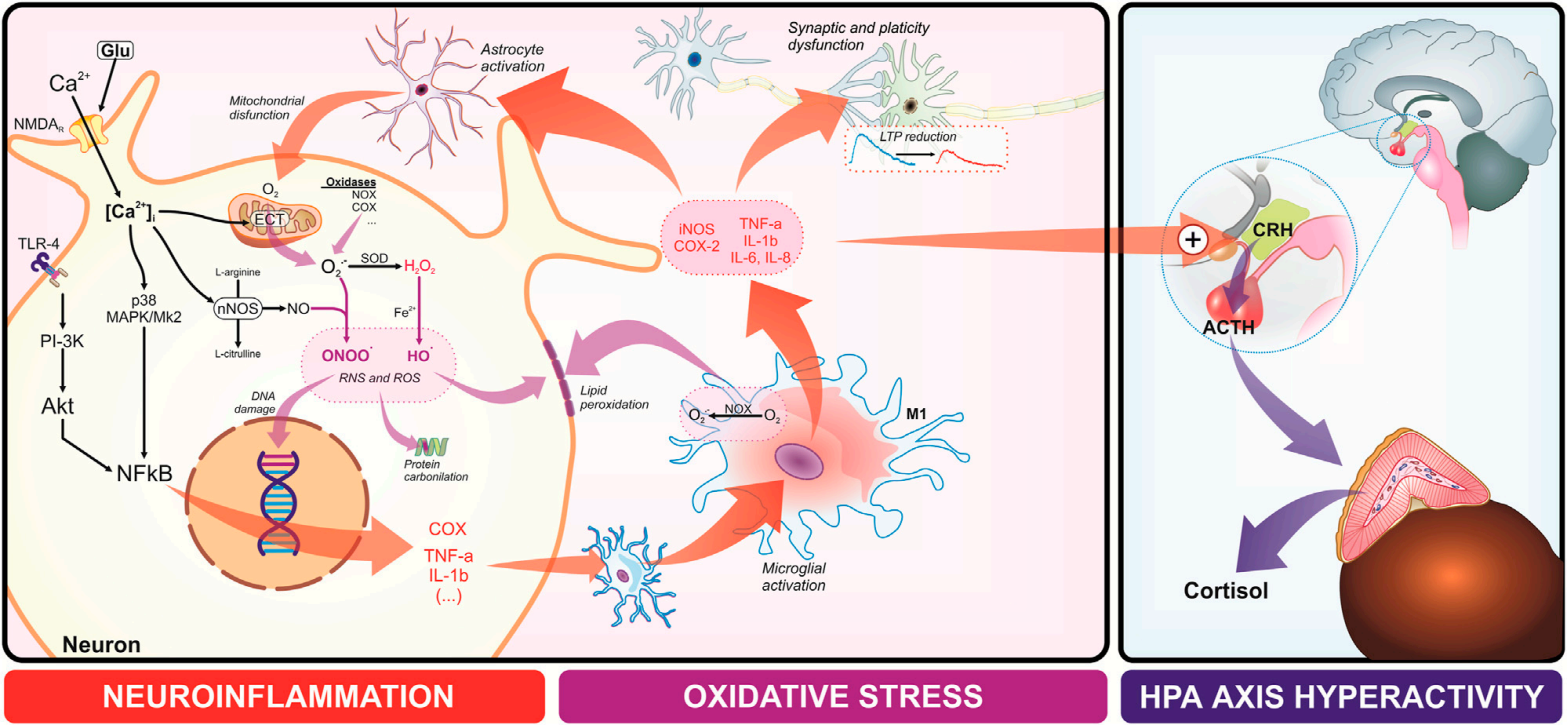
Neuroinflammation, oxidative stress processes on the central nervous system disorders, and the relationship with the hypothalamic-pituitary-adrenal (HPA) axis. In the neuroinflammatory process, an increase in proinflammatory cytokines (e.g., TNF-alpha, Interleukins 1β, 6, 8) triggers glial changes, including the activation of astrocytes and microglia, and leads to synaptic dysfunction due to alterations in long-term potentiation. Additionally, at the intracellular level, heightened neuroinflammation and oxidative stress result in significant calcium influx, which activates inflammatory and oxidative factors, creating a feedback loop between neuroinflammation and oxidative stress.

Numerous studies have confirmed the association between chronic inflammation and depression, anxiety, and other psychiatric disorders, particularly those refractories to conventional medications (Strawbridge et al., 2015; Haroon et al., 2018; Osimo et al., 2020). Similarly, although different neurological disorders are associated with various etiologies, cellular and molecular processes may be shared. Thus, exhaustive evidence suggests that oxidative stress and neuroinflammation play synergistic roles in the development and maintenance of neurological disorders, which in the presence of these disturbances, neurotransmitter dysfunction may occur (Figure 2). Inflammation status displays the overproduction of reactive oxygen species (ROS), favoring oxidative stress, equally that oxidative stress promotes activation of proinflammatory pathways in chronic, degenerative, and neurological conditions (Reuter et al., 2010; Black et al., 2015; Ménard et al., 2016; Maes et al., 2018; Beurel et al., 2020; Wang et al., 2021).

The CNS is particularly vulnerable to oxidative stress due to several specific factors. The brain exhibits the highest oxygen consumption in the body system and a high amount of polyunsaturated fatty acids, that present high susceptibility to lipid peroxidation, a process whereby ROS attaches these lipids. Moreover, the brain presents high concentrations of transition metal ions (i.e., iron ions), self-oxidizing neurotransmitters (i.e., dopamine), and low concentrations of antioxidants (Halliwell, 2012). Therefore, the harmful potential of oxygen, when converted to ROS, finds a favorable environment in the “brain scenario” to oxidative stress.

In neuronal diseases, mitochondrial electron transport chain (ETC) blockade is the primary source of ROS release. Neuroinflammation involves an overproduction of ROS and reactive nitrogen species (RNS) by inflammatory cells, such as astrocytes and microglia, that are activated via pro-inflammatory signals. Astrocyte activation is triggered by microglia-released inflammatory cytokines, amplifying proinflammatory cytokine levels and exacerbating mitochondrial oxidative disorders. Glutamatergic excitotoxicity triggers neuroinflammation, causing excessive calcium influx and subsequent mitochondrial dysfunction and ROS overproduction. Elevated calcium levels activate pro-inflammatory transcription factors, such as nuclear factor-κB (NF-κB), leading to increased expression of pro-inflammatory cytokines, such as interleukin (IL)-1, IL-6, IL-8, and tumor necrosis factor (TNF). Such cascade results in microglial activation, additional ROS and RNS production, and overactivation of phagocytic enzymes, as NADPH oxidase (NOX)-2 and nitric oxide synthase (NOS), generating perilous RNS (i.e., peroxynitrite). These disturbances in neuronal energy metabolism disrupt the blood-brain barrier, facilitating recruitment of peripheral immune cells, tissue damage, and subsequent neuronal death.

**FIGURE 3:**
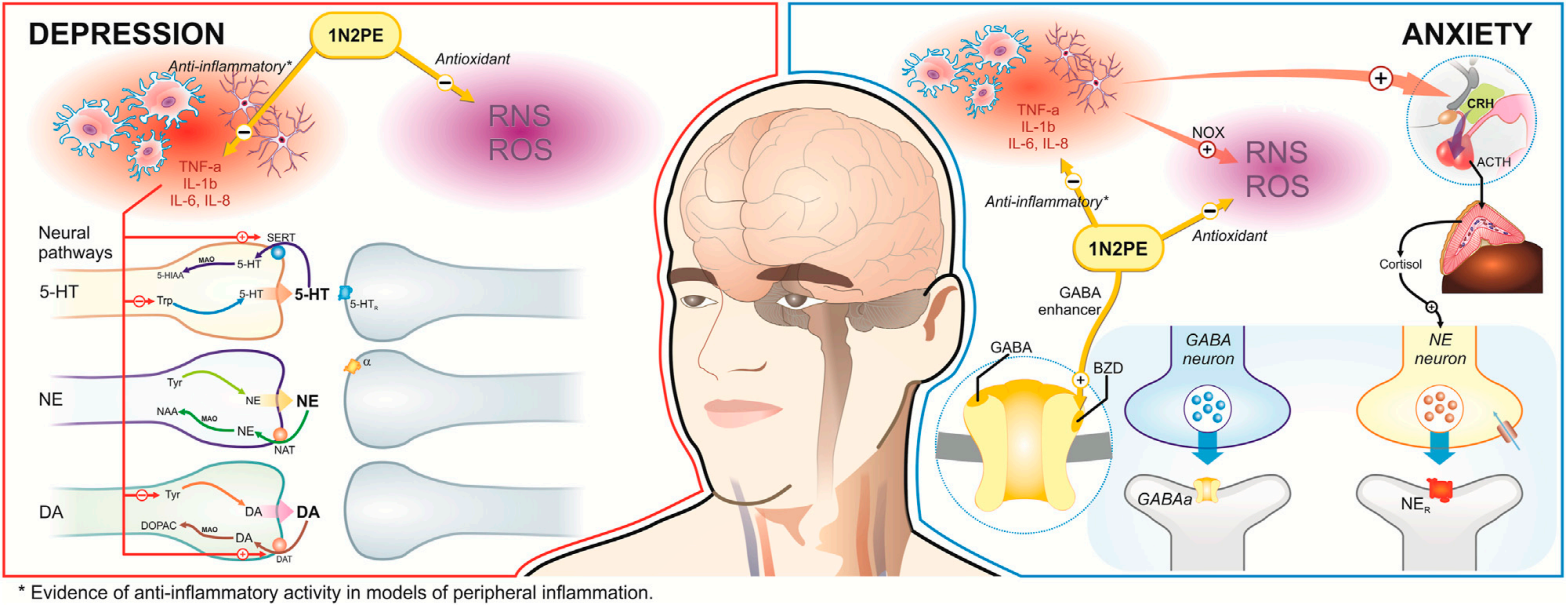
Evidence of 1N2PE biological properties with potential therapeutical benefits for depression and anxiety disorders. 1N2PE demonstrates significant biological properties with potential therapeutic benefits for depression and anxiety disorders. In the context of depression, 1N2PE modulates proinflammatory cytokines and reactive oxygen and nitrogen species, which helps preserve optimal neurotransmission of key monoamines such as dopamine (DA), serotonin (5-HT), and noradrenaline (NE). These neurotransmitters are crucial for regulating mood. For anxiety, 1N2PE not only employs similar mechanisms but also directly interacts with GABA receptors, thereby enhancing GABAergic transmission and contributing to its anxiolytic effects.

Clinical and preclinical evidence suggests that neuroinflammation, for example, is a critical factor that interacts with three other neurobiological correlates of major depressive disorder and anxiety: depletion of cerebral serotonin, dysregulation of the hypothalamic-pituitary-adrenal (HPA) axis, and alteration of neurogenesis on the dentate gyrus of the hippocampus (Thomson and Craighead, 2008; Troubat et al., 2020). The pathophysiological cascade related to neurogenesis disruption appears to be triggered, sustained, and reinforced by chronic inflammatory conditions, which increase peripheral circulating pro-inflammatory markers that can cross the blood-brain barrier and activate microglia, resulting in depression and anxiety (Figure 3). In addition, it can also be a consequence of primary cerebral neuroinflammation, as occurs in neurodegenerative disorders, in which early depressive symptoms generally emerge (Acosta-Rodriguez et al., 2007; Thomson and Craighead, 2008; Troubat et al., 2020).

In addition, cytokines may upregulate the dopamine transporter (DAT) and serotonin transporter (SERT) activity, increasing dopamine and serotonin reuptake, consequently reducing the bioavailability of these neurotransmitters in the synaptic cleft. Therefore, cytokines can affect both the synthesis and reuptake of monoamines, contributing to the onset of depression (Kitagami et al., 2003; Beurel et al., 2020). Microglial activation may also be derived from a direct negative impact of chronic stress (i.e., HPA axis overactivation) on vascular function, which has also been linked to depression (Tan et al., 2019; Troubat et al., 2020; de Jonge et al., 2010; Souza-Junior et al., 2020). Notably, there is a close relationship between inflammation, HPA axis disruption, and autonomic dysfunction. Although depression and hypertension are independent disorders, both are established as risk factors for brain abnormalities (Gianaros et al., 2007).

According to theories postulated in the pathophysiology of depression, the potential antidepressant activity of 1N2PE has been associated with its properties of acting on multiple targets. Firstly, the molecule can cross the blood-brain barrier and permeate the CNS to exert its action (Oyemitan et al., 2013). In addition, the evidence suggests that the 1N2PE exhibits anti-inflammatory activity on the brain, which may protect or mitigate the depression state (Oyemitan et al., 2013). In fact, in classical models of anti-inflammatory activity evaluation, 1N2PE elicited a dose-dependent effect through interaction with prostaglandin-H synthase (PGHS), also called COX-1 (Anderson, 2018). Also, the 1N2PE antioxidant property was first demonstrated through its ability to scavenge 1,1-diphenyl-2-picrylhydrazyl (DPPH) radicals *in vitro* (da Silva et al., 2007).

Recently, our group demonstrated that *Aniba canelilla* essential oil, constituted of ∼80% of NPE, elicits antioxidant properties in a murine model, evidencing its ability to increase the total antioxidant capacity and glutathione (GSH) levels, in addition to reducing lipid peroxidation (Cardoso et al., 2022). We also demonstrated its ability to inhibit leukocyte migration and plasma leakage (Cardoso et al., 2022), which reflects a property with the potential to interfere with leukocyte migration to the brain in the neuroinflammatory process. Finally, studies have postulated that 1N2PE directly affects vascular smooth muscle, inducing relaxation, which deserves further investigations in cerebral vascular diseases (Lahlou et al., 2005; de Siqueira et al., 2010; Interaminense et al., 2011; Interaminense et al., 2013). All these pieces together suggest that behavioral and molecular assays must be designed to explore these hypotheses and measure the impact of 1N2PE on depression.

It is well known that anxiety disturbance, such as obsessive-compulsive and panic disorders, has been linked to oxidative stress (Bouayed et al., 2009; Salim, 2014; Wang et al., 2022). The induction of oxidative stress in experimental models elicited anxiety behavior and a decrease in two antioxidant enzymes (i.e., glutathione reductase and glyoxalase) in the cortex, amygdala, and hippocampus (Hovatta et al., 2005). Microglial activation associated with ROS release by NADPH oxidase (NOX) activity has been associated with anxiety behaviour. NOX inhibition improves anxiety-like behavior in experimental models of behavioral and pathological isolation-inducing anxiety (Schiavone et al., 2009). According to the pathophysiologic mechanism of anxiety discussed, scarce studies point that the 1N2PE emerges as a potential compound in anxiety therapeutics. The elevated plus maze test, a standard model to assess rodent anxiety-like profiles, was employed to detect the effects of 1N2PE. The authors found that 5 and 20 mg/kg (i.p.) of 1N2PE present anxiolytic activity. According to our hypothesis, these outcomes are likely associated with the anti-inflammatory and antioxidant properties of 1N2PE.

**FIGURE 4:**
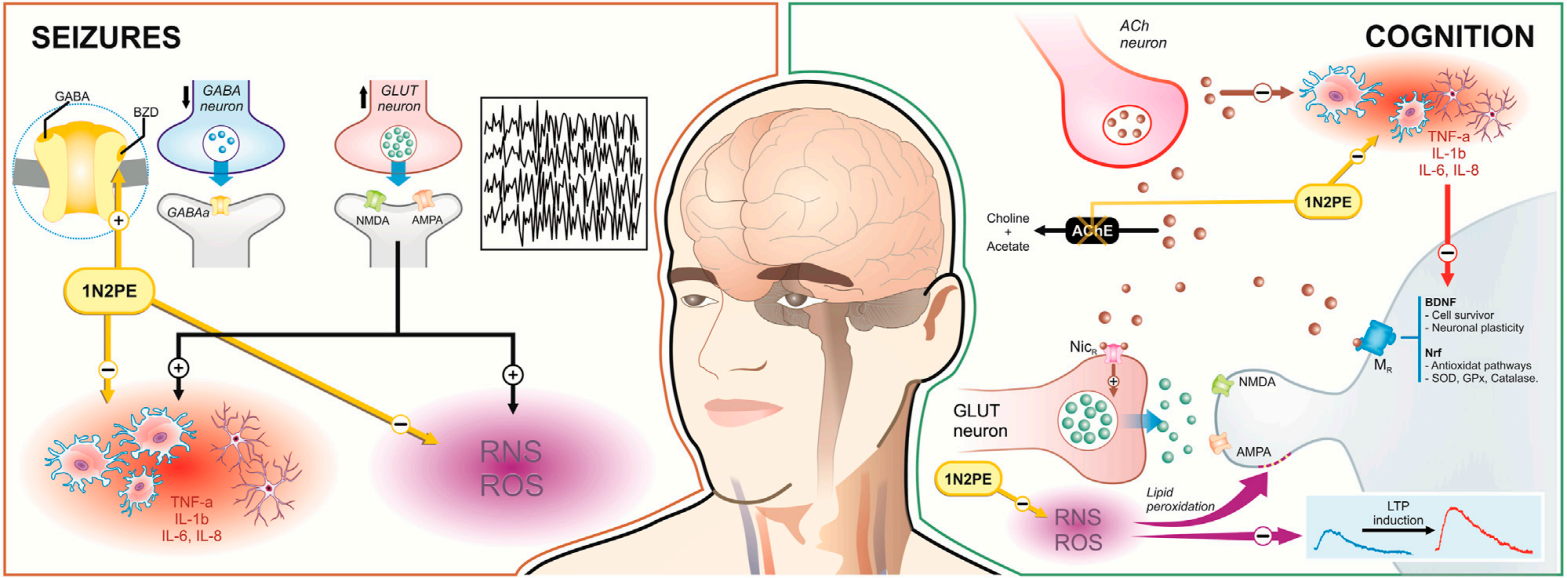
Evidence of 1N2PE biological properties with potential therapeutical benefits for seizures and cognition. For seizures, 1N2PE works through a synergistic mechanism involving direct action on GABAergic receptors and modulation of neuroinflammation and oxidative stress, which helps to attenuate seizure episodes. In terms of cognitive function, 1N2PE significantly inhibits the acetylcholinesterase enzyme, thereby increasing acetylcholine availability. Additionally, by mitigating neuroinflammation, 1N2PE may have an indirect positive effect on brain-derived neurotrophic factor (BDNF), as proinflammatory cytokines typically suppress BDNF levels.

Regarding neurological disorders, the anticonvulsant effects of 1N2PE have been documented. Epilepsy is a neurological disorder affecting millions worldwide, with seizures as its hallmark clinical manifestation (Anwar et al., 2020. While the precise mechanism of seizures remains incompletely understood, abnormal electrical discharges in the brain constitute the primary pathophysiological process. Although approximately 50% of seizures exhibit an unknown aetiology, it is well-defined that atypical excitability and synchronous neuronal spikes in brain regions, caused by extrinsic stimulus (i.e., infection, drug use, metabolic disorders, neurodegenerative diseases, etc.) or congenital factors arise (Falco-Walter, 2020). Considering the different aetiologies for seizures, generally occurs the loss of GABAergic signalling, including interneurons, and degeneration of excitatory cells, affecting glutamate homeostasis, which leads to regional reorganization of the network in brain structures and abnormal electrical input. The astrocytes also negatively contribute to the release of cytokines and hyperactivation of microglia, which in turn elicits the negative modulation of excitatory neurotransmitter reuptake and inhibitory channels, inducing neuronal hyperexcitability (Vera-González, 2022). In infectious aetiology, for example, immunologic and pro-inflammatory signalling emerge, eliciting recurrent neuronal hyperexcitability and seizures (Vera-González, 2022). Therefore, inflammatory cascades, with activation of IL-1, IL-1β, TNF-α, and cyclooxygenase-2 (COX-2), among other inflammatory mediators, which also generate oxidative phenomena, play a pivotal role in seizures (Xu et al., 2013) (Figure 4).

In this scenario, the anticonvulsant effects of 1N2PE have been documented. Oyemitan and colleagues (2013) found that 1N2PE exerts anti-seizure effects likely through a GABAergic pathway, demonstrated through the administration of flumazenil, a GABAA receptor antagonist, which blocked 1N2PE anti-seizure effects. This finding indicates the 1N2PE potential to modulate the GABAergic system, specifically the benzodiazepine site on the GABAA receptor complex. Neuroinflammation significantly contributes to seizures, although the causal relationship between seizures and neuroinflammation remains poorly understood. It is plausible that a bidirectional relationship exists between the two phenomena. Seizures may induce neuroinflammation, while neuroinflammation may exacerbate seizure activity. Notably, seizures promote glutamatergic hyperactivation, initiating cascades of cell death pathways, oxidative stress, and inflammation. The promising anticonvulsant effects of 1N2PE appear intricately linked to its modulation of the GABAergic pathway. However, we hypothesize that 1N2PE may also attenuate neuroinflammatory and oxidative pathways in seizures.

Aligning with this, cognitive dysfunction also represents a multifactorial disturbance. Chronic exposure to extrinsic factors (i.e., physical, psychological, chemical, and environmental stressor stimuli) and intrinsic conditions (i.e., uncommon protein aggregation, co-morbidities, autoimmune disorders, etc.) elicit neuroinflammation, oxidative stress, and mitochondrial impairment, which leads to synaptopathy, interfering in neurotransmission and cognitive processes (Echeverria et al., 2023). Persistent neuroinflammatory and oxidative conditions modify the expression and structure of glutamatergic receptor subunits, impairing synaptic plasticity (Di Filippo et al., 2013). On the other side, natural aging also induces brain cell loss with consequent synaptic network prejudice. An orchestrated long-term harmful occurrence displayed by the immune response produces neuroinflammation, oxidative unbalance, reduced neurotransmission (i.e., glutamatergic, cholinergic, serotoninergic, and dopaminergic traits), decreased neurogenesis, and mitochondrial disturbance, which leads to reduction of synaptic plasticity and strength, and cognitive deficits (Echeverria et al., 2023). In resume, aging, co-morbidities, traumatic experiences, stressor stimuli, neurodegenerative and psychiatric disorders, and other events that negatively alter the CNS could produce a significant degree of pro-inflammatory mediators, reducing the neurotransmitter signaling, long-term potentiation and long-term depression (LTP/LTD) stimulation, neurogenesis, and synaptic plasticity, resulting in cognitive impairment (Bourgognon and Cavanagh, 2020) (Figure 4).

Recently, we showed that *Aniba canelilla* essential oil with 1N2PE isolated at 76.2% and concentrated to 99.4% by chromatography column reversed cognitive disturbances through the cholinergic-related hypothesis, restoring learning and memory in a rodent scopolamine-induced cognitive damage model (de Campos et al., 2023). This study was the first work reporting the potential mnemonic properties of this compound in an *in vivo* model. Previous research has indicated that 1N2PE strongly inhibits acetylcholinesterase, the enzyme responsible for degrading acetylcholine, increasing the acetylcholine availability in the synaptic cleft, which might improve inhibitory/excitatory induction on crucial brain regions, trigger neurotransmission, stimulate neurotrophic factors expression, and mitigate neuroinflammation and oxidative damage (Silva et al., 2014; Echeverria et al., 2023). In addition, the potential anti-inflammatory and antioxidant activities of 1N2PE could potentiate the cholinergic benefits effects, displaying a synergistic effect on cognitive dysfunction (Figure 4). Notably, those hypotheses deserve further investigation.

The specific pathophysiologic mechanisms related to CNS diseases provide potential molecular targets for bioactive compounds. Thus, it seems reasonable to consider that an adjuvant therapy primarily supported by antioxidant and anti-inflammatory molecules, such as 1N2PE, may improve psychiatric and neurological symptoms.

## 7 Conclusion

Several studies have focused on the search for bioactive compounds with neuropharmacological potential for CNS disorders. Within this field, 1N2PE appears as the principal compound in some essential oils that have been poorly investigated. The present work compiled the literature focused on putative CNS activities of 1N2PE since it was first isolated (1959), critically proposing its probable mechanism of action. In this context, it was seen that this organic nitrocompound exhibits a potential therapeutic effect on behavioral disorders, such as depression and anxiety, and neurological disorders, such as dementia and seizures. However, scientific results with 1N2PE are just beginning, underscoring the need for further investigation to unlock its potential as a novel therapeutic agent for neuropsychiatric disorders and neurological impairments.
